# The Role of Primary Care in Service Provision for People with Severe Mental Illness in the United Kingdom

**DOI:** 10.1371/journal.pone.0036468

**Published:** 2012-05-15

**Authors:** Siobhan Reilly, Claire Planner, Mark Hann, David Reeves, Irwin Nazareth, Helen Lester

**Affiliations:** 1 National Institute for Health Research School for Primary Care Research, Health Sciences Research Group, University of Manchester, Manchester, England, United Kingdom; 2 Department of Primary Care and Population Health, University College London School of Medicine, London, England, United Kingdom; 3 School of Health and Population Sciences, University of Birmingham, Birmingham, England, United Kingdom; Tehran University of Medical Sciences, Iran (Islamic Republic of)

## Abstract

**Background:**

Severe mental illness is a serious and potentially life changing set of conditions. This paper describes and analyses patient characteristics and service usage over one year of a representative cohort of people with a diagnosis of severe mental illness across England, including contacts with primary and secondary care and continuity of care.

**Methods and Findings:**

Data were collected from primary care patient notes (n = 1150) by trained nurses from 64 practices in England, covering all service contacts from 1^st^ April 2008 to 31st March 2009. The estimated national rate of patients seen only in primary care in the period was 31.1% (95% C.I. 27.2% to 35.3%) and the rates of schizophrenia and bipolar disorder were 56.8% (95% C.I. 52.3% to 61.2%) and 37.9% (95% C.I. 33.7% to 42.2%). In total, patients had 7,961 consultations within primary care and 1,993 contacts with mental health services (20% of the total). Unemployed individuals diagnosed more recently were more likely to have contact with secondary care. Of those seen in secondary care, 61% had at most two secondary care contacts in the period. Median annual consultation rates with GPs were lower than have been reported for previous years and were only slightly above the general population. Relational continuity in primary care was poor for 21% of patients (Modified Modified Continuity Index  = <0.5), and for almost a third of new referrals to mental health services the primary care record contained no information on the referral outcome.

**Conclusions:**

Primary care is centrally involved in the care of people with serious mental illness, but primary care and cross-boundary continuity is poor for a substantial proportion. Research is needed to determine the impact of poor continuity on patient outcomes, and above all, the impact of new collaborative ways of working at the primary/secondary care interface.

## Introduction

Severe mental illness (a diagnosis of bipolar disorder, schizophrenia or other psychosis) is a serious and potentially life changing set of conditions. Although some people make a full recovery, many develop a lifelong illness [1]. The prevalence of bipolar disorder is about 1–2% of the UK population, although bipolar spectrum disorder may affect as many as 8% [2]. The prevalence of schizophrenia is 0.72% [3]. People with schizophrenia and bipolar disorder die up to 25 years earlier than the general population [4], [5].

Despite its prevalence and poor health outcomes, there has been little recent empirical work on the structure and processes of health care for people with severe mental illness. In the United Kingdom, historically people with severe mental illness are thought to consult primary care practitioners more frequently [6]–[8] and are in contact with primary care services for a longer cumulative time than patients without mental health problems [9], [10]. The most recent and directly relevant study found an annual primary care consultation rate of 13.3 [7]. However that data is now over 15 years old. The importance of continuity of care (see Box S1) for people with severe mental illness is also well recognised though rarely captured [11]–[15].

Previous studies have tended to draw on either databases which have the advantages of large numbers but not granularity, or relatively small scale notes audits with limited power. These factors have led to large differences in, for example, the most basic descriptors of service usage such as locus of care (see Box S1). Somewhere between 18–30 per cent of people with severe mental illness in the UK are described as being seen only in a primary care setting, a variation with considerable implications for health service commissioning and costs [8], [16]–[18].

This paper describes a cross sectional cohort study of the current state of health and health care of people with severe mental illness in England. The aims of this study were to a) identify and describe the health service use, locus of primary and secondary care, relational, cross boundary and informational continuity of care of a large representative cohort of people with a diagnosis of severe mental illness across England; b) to compare these by locus of mental health care and severe mental health diagnosis; and c) explore the factors associated with locus of mental health care and poor continuity of care.

## Results

Slower than anticipated recruitment resulted in the achieved practice sample being smaller than the original target: 64 practices in 51 different Primary Care Trusts across England were recruited. The median practice size was 9,011 [IQR (6,345, 13,757)]: 48 (75%) were training practices. 18% of practice nurses reported that they had some form of mental health training. The median number of patients on practices’ mental health registers was 66 [IQR (43, 105)] and the median number of those eligible for the study was 50 [IQR (26, 71)].

Each practice was provided with a list of random numbers for the purpose of sampling 20 eligible patients from their register. Some smaller practices did not have 20 eligible patients hence data were extracted for a total of 1,150 patients (practice mean = 18; median = 20; range = 8–21).

### Locus of Care

The results presented in the tables provide comparative data on demography, health and medication details for those seen only within primary care and those who were in contact with secondary care mental health services over the period 1/4/2008 – 31/3/2009. The results are further divided between those with schizophrenia and those with bipolar disorder.

Of the total patient sample 30.8% (354 out of 1150) were seen only in primary care over the 12 month period (ie had no evidence for any secondary care contacts); the other 69.2% (796) had at least one secondary care contact during the period. 56.3% (647) of patients had a diagnosis of schizophrenia and 37.7% (433) bipolar disorder. After weighting for practice register sizes and socioeconomic deprivation indices, the estimated national rate of patients seen only in primary care in the period was 31.1% (95% C.I. 27.2% to 35.3%) and the rates of schizophrenia and bipolar disorder were 56.8% (95% C.I. 52.3% to 61.2%) and 37.9% (95% C.I. 33.7% to 42.2%).

### Patient Demographics

The average age of the 1,150 patients was 52.7 years (standard deviation, SD  = 14.7) and 53.1% were male. 13.2% were reported to be non-white, although information on ethnic origin was missing for 17.4%. A third (33.3%) of the sample lived alone and 26.4% lived with their spouse/partner. Just 12.6% were reported to be in employment, but employment status was unknown for 22.8%. The average duration of a patients’ illness was 17.3 years (SD = 12.0; median  = 14.0) (Table 1).

**Table 1:** Demographic characteristics, weighted means and percentages.

	Locus of care - secondary care mental health services			Locus of care - primary care only			All patients	Comparison of loci^4^
SMI diagnosis	Schizophrenia	Bipolar disorder	All diagnoses^1^	Schizophrenia	Bipolar disorder	All diagnoses^1^	All diagnoses^1^	p-value
**N**	461	286	796	186	147	354	1,150	-
**Average age** (years)	49.4	53.7	51.0	56.8	57.8	56.5	52.7	<0.001
**Average duration of illness** (years)	18.6	12.4	16.1	22.4	17.5	19.7	17.3	<0.001
**Average age at diagnosis** (years)	30.8	41.3	34.9	34.4	40.3	36.8	35.5	0.11
**Gender** (%)								0.37^5^
M	57.8	44.2	51.5	61.1	47.6	56.6	53.1	
F	42.3	55.8	48.5	38.9	52.4	43.4	46.9	
**Ethnic group** (%)								0.62^5^
White	63.7	80.4	70.3	63.9	76.3	67.4	69.4	
Non-white	19.0	5.4	13.1	16.9	8.9	13.4	13.2	
Not recorded/missing	17.3	14.2	16.6	19.2	14.8	19.2	17.4	
**Living situation** (%)								0.13^5^
Alone	32.2	41.2	35.2	35.4	22.0	28.9	33.3	
With spouse/partner^2^	21.5	27.9	23.7	21.1	50.4	32.4	26.4	
Group Home^3^	21.0	6.3	15.7	17.2	6.0	14.3	15.3	
Other/missing	25.4	24.6	25.3	26.4	21.6	24.5	25.1	
**Current employment** (%)								<0.001^5^
Employed	9.0	18.0	12.8	7.2	16.9	12.0	12.6	
Unemployed	32.0	25.1	28.4	15.9	4.3	10.4	22.8	
Economically inactive	41.6	38.0	40.5	44.4	42.8	44.7	41.8	
Missing	17.4	18.9	18.3	32.5	35.9	32.9	22.8	

1 Includes an additional 70 patients (49 with secondary care MHS; 21 primary care only): 46 with other psychoses (e.g. psychotic illnesses, non-organic psychotic disorder, schizoaffective disorder) and 24 with no specified diagnosis.

2 May or may not include children.

3 Includes residential/nursing home and sheltered/supported housing.

4 Statistical comparisons between all secondary care and all primary care only patients. *P*-value from weighted linear regression analysis unless otherwise stated.

5 Weighted logistic/multinomial logistic (where more than two categories) regression analysis; omnibus test comparing all levels of the explanatory variable.

Direct comparisons between patients with secondary care contacts and those without did not find any differences in gender, age of diagnosis, ethnic group or living situation (p>0.05 in all cases), but did find that the former group were younger on average (by 5.5 years; t = 5.66; p<0.001) and likely to have been diagnosed more recently (3.6 years on average; t = 4.22; p = <0.001).

### Mental Health Status

Most people were in receipt of prescriptions for mental health medication (89.7%), with 7.7% not in receipt of medication and another 2.6% where this information was not recorded (Table 2). People seen in secondary care had slightly fewer health morbidities on average (mean 1.3 versus 1.5; χ^2^_(1) = _3.71; p = 0.054), but much more likely to have a dual diagnosis (21.8% versus 12.2%; χ^2^_(2) = _8.65; p = 0.013) and to have a greater number of prescribed mental health medications compared to those seen only in primary care (mean 1.9 versus 1.4; χ^2^_(1) = _5.60; p = 0.018).

**Table 2:** Health and medication details, weighted percentages.

	Locus of care - secondary care mental health services			Locus of care - primary care only			All patients	Comparison of loci
SMI diagnosis	Schizophrenia	Bipolar disorder	All diagnoses^1^	Schizophrenia	Bipolar disorder	All diagnoses^1^	All diagnoses^1^	p-value
**N**	461	286	796	186	147	354	1,150	-
**Major health morbidities (%)** 2								0.054^4^
None	33.7	29.4	32.1	30.1	27.6	29.2	31.2	
1	31.9	29.9	31.0	32.2	34.4	32.4	31.4	
2	18.6	22.6	20.6	14.6	13.6	15.5	19.0	
3	11.7	12.8	11.8	11.1	11.0	10.4	11.3	
≥4	4.1	5.3	4.6	12.0	13.4	12.5	7.0	
Mean (median) morbidities	1.2 (1)	1.4 (1)	1.3 (1)	1.4 (1)	1.6 (1)	1.5 (1)	1.3 (1)	
**Dual diagnosis (%)**								0.013^5^
Yes	18.1	27.5	21.8	13.1	11.0	12.2	18.9	
No	61.3	59.3	60.2	62.6	78.0	69.4	63.1	
Not known	20.6	13.2	17.9	24.3	11.1	18.4	18.1	
**Mental health medications (%)**								0.018^4^
None	5.2	3.7	4.4	15.8	13.6	15.1	7.7	
1	34.2	31.3	34.0	45.2	38.9	44.0	37.1	
2	37.2	34.4	35.5	24.2	32.9	26.6	32.7	
3	17.0	20.1	17.9	3.8	7.0	4.9	13.9	
≥4	5.5	8.9	6.8	4.3	4.2	4.2	6.0	
Missing^6^	0.9	1.6	1.4	6.7	3.4	5.2	2.6	
Mean (median) medications	1.9 (2)	2.0 (2)	1.9 (2)	1.5 (1)	1.4 (1)	1.4 (1)	1.8 (2)	
**Type of medication (%)** 3								
Atypical antipsychotic	63.7	27.4	49.3	34.2	22.4	30.7	43.5	<0.001^7^
Antidepressant med	31.8	46.8	37.4	17.8	38.5	26.0	33.8	0.010^7^
Conventional antipsychotic	27.0	17.8	22.8	32.4	18.6	25.5	23.7	0.54^7^
Bipolar disorder med	8.4	54.6	25.6	2.7	41.7	18.2	23.3	0.08^7^
Anti-anxiety med	8.2	9.3	9.1	3.5	5.5	4.4	7.6	0.010^7^
Other med	24.8	15.6	21.6	15.6	7.4	11.8	18.5	<0.001^7^

1 Includes an additional 70 patients (49 with secondary care MHS contact; 21 primary care only).

2 Total out of diabetes, asthma, chronic obstructive pulmonary disorder, epilepsy, hypertension, stroke, thyroid, ischaemic heart disease, heart failure, chronic kidney disease, depression, learning disability, hearing problems, rheumatoid arthritis, cancer, osteoarthritis, obesity, visual problems, and 18 other less frequently recorded long-term conditions.

3 Patients can be receiving more than one type of medication, therefore percentages can add up to more than 100%.

4 Weighted poisson regression analysis.

5 Weighted multinomial logistic regression analysis; omnibus test comparing all three levels.

6 Missing cases were included in the analysis using regression imputation (on age, gender, number of morbidities and loci of care).

7 Weighted logistic regression analysis comparing rates of patients prescribed each medication.

### Health Service Use

In total, patients had 7,961 consultations within primary care and 1,993 contacts with mental health services (representing 20% of total contacts) during the period (Table 3). Most consultations in primary care were with a GP (62%) or a nurse (28%). Most of the secondary care consultations were with a psychiatrist (67%).

**Table 3:** Contacts in primary and secondary care, unweighted counts.

Locus of care	Contact with	Number of contacts	% of contacts
**Primary care only**	GP	4,946	62
	Nurse	2,218	28
	Other professional	797	10
	Total	7,961	80% of all contacts
**Secondary care**	Psychiatrist	1,338	67
	CPN	168	8
	Other professional	487	24
	Total	1,993	20% of all contacts
**Primary and secondary care**	Total	9,954	100%

Most patients had one or more consultations with a general practitioner during the year (88.7%) (Table 4). The mean consultation rate for all 1,150 patients was 4.3 and was higher for those seen in secondary care (4.6 compared to 3.7 for those seen in primary care only; χ^2^_(1) = _7.0; p = 0.008). Secondary care patients also saw a greater number of different GPs on average (1.9 versus 1.5; χ^2^_(1) = _6.35; p = 0.012). Almost two thirds of patients had one or more consultations with a practice nurse during the year (59.1%). The mean nurse consultation rate for all 1,150 patients was 2.1.

**Table 4:** GP face to face consultations and relational continuity of care, weighted percentages.

	Locus of care - secondary care mental health services			Locus of care - primary care only			All patients	Comparison of loci
SMI diagnosis	Schizophrenia	Bipolar disorder	All diagnoses^1^	Schizophrenia	Bipolar disorder	All diagnoses^1^	All diagnoses^1^	p-value
**N**	461	286	796	186	147	354	1,150	
**Contacts with a GP (%)**								0.008^4^
None	11.5	9.4	10.7	12.1	11.1	12.7	11.3	
1/2	27.9	19.5	24.5	31.2	27.1	28.3	25.6	
3/4	24.7	22.2	23.8	21.0	26.6	24.5	24.0	
5/6	12.9	16.6	15.1	18.1	11.4	14.8	15.0	
≥7	23.0	32.2	26.0	17.7	23.9	19.8	24.1	
Mean (median) contacts	4.0 (3)	5.3 (4)	4.6 (4)	3.4 (3)	4.2 (3)	3.7 (3)	4.3 (3)	
**Number of GPs seen (%)**								0.012^4^
None	11.5	9.4	10.7	12.1	11.1	12.7	11.3	
1	45.1	43.6	43.8	55.2	42.3	48.7	45.3	
2	13.8	18.1	15.7	15.1	18.9	17.8	16.3	
3	16.6	11.7	14.8	12.8	22.6	16.0	15.2	
≥4	13.1	17.2	15.1	4.8	5.1	4.9	12.0	
Mean (median) GPs seen	1.8 (1)	1.9 (1)	1.9 (1)	1.5 (1)	1.7 (1)	1.5 (1)	1.8 (1)	
**Reason for contact (%)** 2								
Physical health problem	66.3	67.3	66.9	70.2	69.8	68.9	67.5	0.71^5^
Mental health problem	40.9	57.5	46.9	20.0	42.8	29.7	41.5	<0.001^5^
Medication review	37.8	46.2	41.2	46.5	39.0	44.1	42.1	0.56^5^
QOF review	35.3	29.7	34.3	45.9	39.4	42.0	36.7	0.046^5^
Health education	14.6	13.3	14.9	29.7	14.9	22.0	17.1	0.054^5^
Repeat prescription	12.4	9.8	11.6	8.3	11.3	8.9	10.8	0.28^5^
Other/Unknown reason	11.9	10.9	12.1	19.9	13.2	17.9	13.9	0.18^5^
**Poor continuity of care** 3 **(%, (N))**								
Overall	21.5 (265)	16.4 (211)	20.7 (507)	19.0 (90)	23.4 (90)	20.2 (190)	20.6 (697)	0.86^6^
3 contacts	15.2 (70)	10.4 (30)	14.9 (105)	11.2 (29)	10.5 (25)	11.2 (56)	13.6 (161)	-
4 contacts	44.8 (45)	24.2 (32)	37.2 (84)	12.4 (13)	46.2 (14)	19.3 (29)	32.1 (113)	-
5 contacts	35.7 (35)	41.7 (27)	36.6 (67)	59.0 (17)	73.2 (13)	62.0 (31)	45.3 (98)	-
6 contacts	28.5 (28)	3.5 (25)	25.6 (55)	18.7 (12)	0.0 (3)	16.6 (15)	23.3 (70)	-
≥7 contacts	8.6 (87)	12.4 (97)	10.4 (196)	6.0 (19)	8.4 (35)	8.3 (59)	9.9 (255)	-

1 Includes an additional 70 patients (49 with secondary care MHS contact; 21 primary care only).

2 The % of patients to whom each reason applies. Percentages can add to more than 100%. Reasons applying to <5% of all patients have been excluded, these include sick note requests, blood tests, vaccinations, medication injection, and family, housing, employment and financial problems.

3 Excludes 453 patients with less than three GP contacts (289 with secondary care MHS contact and 164 primary care only) for whom continuity could not be assessed.

4 Weighted poisson regression analysis.

5 Weighted logistic regression analysis comparing rates of reports of each reason.

6 Weighted logistic regression analysis comparing overall rates of poor continuity.

Physical health problems were cited more frequently than mental health problems as reasons for contacts regardless of locus of care or mental illness diagnosis (67.5% of all patients reported consulting a GP for a physical problem; 41.5% for a mental health problem). However, patients in contact with secondary mental health services were more likely to consult a GP for a mental health reason compared to those seen only in primary care (46.9% vs. 29.7% respectively; χ^2^_(1)_ = 23.91; p<0.001). Health education was a component in 17.1% of all consultations, and was borderline significantly different between loci of care (14.9% versus 22.0%; χ^2^_(1)_ = 3.70; p = 0.054).

Of the 69% of patients seen in secondary care, 61% had at most two contacts over the year with secondary mental health services (Table 5). Almost 12% of this cohort had a mental health admission (8% voluntary; 4% compulsory) during the 12 months. Most patients (96%) were seen by a community mental health team, outpatient psychiatry, rehabilitation/ recovery, or other non-intensive teams. 6% were in contact with home treatment teams/ crisis resolution, assertive community treatment, early intervention services or forensic services or outreach services.

**Table 5:** Contacts with mental health professionals, weighted percentages.

		Locus of care - secondary care mental health services		
SMI diagnosis		Schizophrenia	Bipolar disorder	All diagnoses^1^
**N**		461	286	796
**Number of contacts in secondary care (%)**	Not recorded	11.4	16.7	13.4
	1/2	48.3	44.2	47.5
	3/4	29.3	22.1	26.1
	5/6	6.8	7.7	7.1
	≥7	4.2	9.3	6.0
	Mean (median) contacts	2.4 (2)	2.7 (2)	2.5 (2)
**Service type (%)**	High intensity^2^	5.9	4.6	5.6
	Other^3^	94.1	95.4	94.4
**Contact with (% of patients)** 4	Consultant psychiatrist	46.1	48.5	46.7
	Staff grade psychiatrist	20.4	22.1	20.6
	CPN	10.3	11.1	10.4
	Other MH professional	17.7	13.6	15.8
	Unknown MH professional	24.1	25.3	25.6
**Contact with (% of all contacts)** 5	Consultant psychiatrist	45.6	52.6	48.5
	Staff grade psychiatrist	22.3	21.7	22.0
	CPN	8.2	6.1	7.3
	Other MH professional	20.5	14.2	17.9
	Unknown MH professional	3.4	5.5	4.2

1 Includes an additional 49 patients with other psychoses e.g. psychotic illnesses, non-organic psychotic disorder, schizoaffective disorder or with no specified diagnosis.

2 Patients who have been in contact with Home Treatment Teams/ Crisis Resolution, Assertive Community Treatment, Early Intervention Services, Forensic Services or Outreach Services.

3 Patients in contact with CMHT, Outpatient Psychiatry, Rehabilitation/ Recovery, Family Therapy, Inpatient Detox, Psychology, Shared Care for Substance Misuse. Includes 134 patients with no contact data recorded and 86 in contact with an ‘unidentified’ service, but with evidence of a care coordinator or a psychiatrist.

4 Can add up to more than 100%.

5 Contacts as a % of all contacts (total contacts: schizophrenia patients 1,084; bipolar patients 808; all diagnoses 1,993).

### Continuity of Primary Care

Calculation of relational continuity of primary care was restricted to patients with a minimum of three GP contacts (n = 697) (Table 4). One-fifth (20.6%) of these patients had poor continuity. Patients who had five GP contacts over the year were the most likely to have poor continuity (45.3%), whilst patients with seven or more GP contacts were the least likely (9.9%).

There was no significant difference in the rates of poor relational continuity between patients who were seen in primary care alone and patients who were also seen in secondary care (p = 0.86).

Informational continuity, the timely availability of information, also appeared to be poor. Data relating to all patients who had a new referral to a mental health service over the year (n = 266) indicated that no information was recorded in primary care about the outcome of the referral for 28.7% of patients, 5.1% of patients were not seen by the mental health services and a further 1.7% were seen according to free text notes, but no documentation had been received.

Cross-boundary continuity, which we have measured as transitions and fragmentations in care, was poor for a substantial proportion of patients. Of those who were discharged from a mental health service in the period of study (n = 111), 8.1% were either lost to follow up for no apparent reason or did not attend the appointment and a further 14.9% did not have a reason recorded for their discharge.

### Patient and Practice Predictors of Locus of Care

Univariate and multivariate logistic analysis identified a number of patient and practice characteristics associated with being seen by secondary care mental services (Table 6). Examined individually, factors predictive of being seen in secondary care were: younger age (p<0.001); fewer years since diagnosis (p<0.001); a dual diagnosis (p = 0.013); and economic activity status (p<0.001), in particular being unemployed. After multivariate adjustment only years since diagnosis (p = 0.006) and economic activity (p<0.001) remained significant.

**Table 6:** Summary of weighted univariate and multivariate regressions for locus of care and poor relational continuity of care.

		Locus of care secondary care mental health services (N = 1,150)				Poor relational continuity of care (N = 697^1^)			
		Univariate logistic regression		Multivariate logistic regression		Univariate logistic regression		Multivariate logistic regression	
		Odds Ratio (95% CI)	p-value^2^	Odds Ratio (95% CI)	p-value^2^	Odds Ratio (95% CI)	p-value^2^	Odds Ratio (95% CI)	p-value^2^
**Age in years** 3		0.77 (0.71, 0.84)	<0.001	0.88 (0.75, 1.04)	0.13	1.01 (0.80, 1.28)	0.91		
**Years since diagnosis** 3		0.78 (0.71, 0.87)	<0.001	0.85 (0.76, 0.96)	0.006	1.01 (0.83, 1.24)	0.90		
**Gender**	Male	Reference	0.37			Reference	0.072	1.42 (0.91, 2.22)	0.12
	Female	1.23 (0.78, 1.92)				1.49 (0.97, 2.29)			
**Ethnicity**	White	Reference	0.62			Reference	0.089	Reference	0.16
	Minority	0.94 (0.58, 1.51)				0.46 (0.18, 1.19)		0.48 (0.18, 1.26)	
	Not known	0.83 (0.52, 1.32)				1.74 (0.90, 3.35)		1.49 (0.73, 3.05)	
**Living status**	Alone	Reference	0.13	Reference	0.71	Reference	0.47		
	With spouse	0.60 (0.39, 0.93)		0.81 (0.54, 1.21)		1.60 (0.88, 2.92)			
	Group Home	0.90 (0.50, 1.64)		1.09 (0.55, 2.14)		1.30 (0.66, 2.54)			
	Other/not known	0.85 (0.52, 1.39)		1.01 (0.56, 1.82)		1.63 (0.75, 3.56)			
**Economic activity**	Employed	Reference	<0.001	Reference	<0.001	Reference	0.047	Reference	0.40
	Unemployed	2.57 (1.71, 3.88)		2.54 (1.71, 3.75)		0.67 (0.29, 1.53)		0.71 (0.31, 1.60)	
	Inactive	0.85 (0.56, 1.30)		1.11 (0.70, 1.77)		0.77 (0.42, 1.43)		0.84 (0.42, 1.70)	
	Not known	0.52 (0.28, 0.96)		0.60 (0.34, 1.07)		1.50 (0.81, 2.78)		1.22 (0.65, 2.29)	
**Major health morbidities**	None	Reference	0.12	Reference	0.23	Reference	0.28		
	1	0.87 (0.66, 1.15)		0.86 (0.61, 1.22)		0.55 (0.29, 1.01)			
	2	1.20 (0.70, 2.01)		1.31 (0.76, 2.26)		0.86 (0.44, 1.66)			
	3	1.03 (0.59, 1.81)		1.23 (0.779, 1.98)		0.63 (0.24, 1.66)			
	4 or more	0.33 (0.14, 0.78)		0.45 (0.17, 1.21)		1.00 (0.47, 2.14)			
**SMI diagnosis**	Schizophrenia	Reference	0.22			Reference	0.21		
	Bipolar	0.89 (0.65, 1.24)				0.86 (0.57, 1.30)			
	Other/not known	0.71 (0.47, 1.10)				1.94 (0.86, 4.36)			
**Dual diagnosis**	No	Reference	0.013	Reference	0.18	Reference	0.60		
	Yes	2.06 (1.27, 3.33)		1.58 (0.96, 2.59)		1.19 (0.78, 1.82)			
	Not known	1.13 (0.73, 1.74)		1.21 (0.80, 1.82)		0.83 (0.40, 1.73)			
**Number of GP contacts**	None	Reference	0.45			Not applicable^1^			
	1 or 2	1.03 (0.59, 1.78)				Not applicable^1^			
	3 or 4	1.15 (0.62, 2.15)				Reference	0.003	Reference	0.017
	5 or 6	1.21 (0.61, 2.41)				2.05 (1.28, 3.30)		2.03 (1.13, 3.63)	
	7 or more	1.56 (0.80, 3.05)				0.40 (0.19, 0.83)		0.38 (0.21, 0.68)	
**Secondary care contact**	No	Not applicable				Reference	0.86		
	Yes	Not applicable				1.03 (0.73, 1.45)			
**Practice list size** 4		1.00 (0.95, 1.04)	0.87			1.08 (1.02, 1.14)	0.009	1.06 (0.99, 1.15)	0.084
**IMD 2007** 3		1.16 (1.00, 1.40)	0.11	1.06 (0.93, 1.21)	0.37	0.92 (0.68, 1.27)	0.64		

1 Patients with less than three GP contacts were excluded from the analyses for continuity of care.

2 P-values relate to omnibus tests comparing all levels of each explanatory variable.

3 Odds Ratio for a 10 unit increase in the explanatory variable; e.g. 10 years. A higher ‘score’ on the index of multiple deprivation (IMD 2007) indicates a more deprived area.

4 Odds Ratio for an increase of 1,000 patients in the practice total register.

### Patient and Practice Predictors of Poor Relational Continuity

In both univariate and multivariate models the strongest single predictor of poor continuity of primary care was number of GP contacts (Table 6; p = 0.003 and p = 0.017 respectively): patients with 5 to 6 GP contacts were most likely to have poor relational continuity, whilst those with 7 or more contacts were least likely. Although poor continuity was also associated with practice size (p = 0.009) and economic activity status (p = 0.047) in the univariate models, both relationships ceased to be significant under multivariate analysis.

## Discussion

### Summary of Findings

This study suggests that primary care is centrally involved in the care of people with serious mental illness. Nearly a third of patients with a current diagnosis of psychosis were seen only in primary care and of the two thirds of people seen in secondary care, 61% had at most two secondary care contacts recorded in their primary care notes. Annual consultation rates with GPs in primary care were far lower than previously reported, although still slightly higher than that of the general population [19]. Practice nurses did not appear to be centrally involved in care and health education was not a common feature of consultations.

For patients in contact with secondary mental health services, relational continuity in primary care was far from good and informational and cross boundary continuity of care also appeared to be poor. Rates of poor relational continuity rose as primary care contacts increased to 5 contacts - at which point nearly two-thirds of this group had poor relational continuity - but then decreased again. However, this decrease is a function of the limited number of GPs available to consult with at a practice: once all GPs have been consulted at least once, additional contacts can only improve the MMCI score.

### Limitations

Although we did not achieve the pre-study target of 1,600 patients in 80 practices, the 95% confidence interval around our main outcome (the percentage of patients managed entirely in primary care) was nonetheless very close to the desired precision of plus/minus 4%.

This was a cross sectional study so any associations in the data are necessarily associative rather than causal. However our design and methodology enabled detailed patient level data collection of continuity and also locus of care, data that would have been almost impossible to replicate using national primary care databases [20]. The data collection process was very time consuming for practice nurses but accuracy was increased through a detailed study manual, ongoing telephone and email support and feedback from the study team as well as regular checks and follow up of missing data.

Although there was a good geographical spread of practices, GPRF practices are over-representative of large practices in less deprived areas and only twelve of the recruited practices had a list size below the national median. We compensated for this by applying sampling weights to produce approximate nationally representative results. The demographic details of the patient sample were however similar to other contemporaneous cohort studies of people with serious mental illness in terms of gender and employment rates [21]. In addition, unweighted and weighted estimates of the percentage of patients seen in primary care differed by only 0.3%, indicating that the estimate is stable. However, it is possible that the mental health services contact data in primary care notes under represented actual contacts with secondary care.

### Comparisons with Previous Work

Research over the last twenty years has suggested that many general practitioners feel that, in contrast to patients with potentially complex illnesses such as diabetes or heart failure, holistic care of patients with psychosis is beyond their remit [22], [23]. The majority regard themselves as simply involved in the monitoring and treatment of physical illness and prescribing for mental illness [22]–[24]. However this study suggests that on a per annum basis about a third of people with severe mental illness are not seen in secondary care (a figure similar to that in the largest previous survey) and consult primary care for ongoing mental health reasons far more than previously recognised. Kendrick and colleagues for example found that only about 32% of consultations were focused on mental health issues [8]. In contrast to previous work, consultation rates for this population appear to be far lower than the rates of 13 to 14 per annum reported in the mid-1990s [6], [7] and are currently only slightly higher than the general population: the median (IQR) practice surgery consultation rate for this population was 3 (2–6), whereas the median consultation rate with a General Practitioner for the general population has remained more stable over time: 2.7 (2.2–3.1) in 1995, rising to 2.8 (2.5–3.2) in 2008 [19]. The median consultation rates for the general population with practice nurses was 1.8 (1.3–2.3) in 2008 [19], somewhat higher than in this study population (a median of 1 (0–3)).

Relational continuity is jointly produced by the system, the individual provider, and the patient. Problems can occur when there are barriers at any of these levels (e.g. an appointment system that makes personal continuity difficult) or if the patient is not an effective negotiator or is disadvantaged, for example because of their social circumstances or ethnic group [25]. People with severe mental illness value continuity of care but this study suggests that for a substantial minority this is not currently being achieved in primary care. Poor informational continuity was also present in almost a third of all new referrals – a finding consistent with Bindman’s study over 15 years ago [23], which is disappointing given the intervening evidence of its importance. A recent survey undertaken at the same time as this study found that almost a third of patients were seen by a community psychiatric nurse [26], compared to ten per cent of our sample. However this discrepancy is likely to represent further informational discontinuity between community mental health services and primary care. Outpatient doctors routinely write to General Practitioners after each consultation whereas community key workers who see patients more frequently do not [23].

### Implications for Policy, Practice and Research

This study provides an evidence base on the locus and type of care provided to a large random sample of people with severe mental illness across England. Our data suggest that those only in contact with primary care may have fewer mental health needs (assessed by the proxy measures of medication use and dual diagnosis) and just under half of patients seen in secondary care were receiving minimal support with poor cross boundary and informational continuity between sectors. The need to reduce health service costs is an important principle in many health systems internationally and this data may support policy makers and health professionals towards discharging patients with severe mental illness into a primary care environment. Consultation rates in primary care were also lower than expected, again suggesting that primary care would not be overwhelmed by a new workload. Discharge to primary care alone might also be more feasible and safer if patients were subsequently followed up and supported through a system of collaborative care in that sector [27].

Implications for primary care also include the need for a greater focus on health education. Evidence from the United States suggests that while patients with schizophrenia are less likely to report physical symptoms spontaneously, systematic questioning is effective in uncovering physical illness in this group [28]. Practice nurses, a key workforce in terms of health education, also appear to be underutilised, although more training may need to be made available before they feel comfortable in managing patients with multiple morbidities that include psychosis.

Further research is needed on the impact of poor continuity on patient outcomes, and above all, the impact of new collaborative ways of working at the primary/secondary care interface.

## Methods

### Ethics Statement

The research team obtained ethical approval for the study from the West Midlands Research Ethics Committee, REC reference number 08/H1208/15.

### Sampling Frame and Participants

The study was powered to yield an acceptably precise estimate of the percentage of patients with severe mental illness receiving care in primary care alone. We performed a range of sample size calculations and selected a design with 80 practices and 20 randomly selected eligible patients from each practice, that would estimate this percentage with an error of at most ±4% (95% confidence limit; to be conservative we assumed a true rate of 35% (5% above the previous highest estimate) and an intracluster correlation coefficient of 0.1). To recruit practices we invited all 716 MRC General Practice Research Framework (GPRF) practices in England to express interest in participating. Patient inclusion criteria were: i) diagnosis of schizophrenia, bipolar affective disorder or other psychoses; ii) aged 18 years before 1st April, 2007; iii) added to the Quality Outcomes Framework practice mental health register before 1st April, 2007; and iv) living in the community.

### Concepts Measured and Data Extraction Proforma (See Box S1 and S2)

Data were collected from primary care patient notes by nurses, employed by the practices, between March and September 2010. Data collection procedures were specified in a detailed study manual which was piloted in three practices. CP was also available to address queries by email and telephone and undertake quality checks throughout the study. Nurses reported that they were confident in their answers to all or most of the questionnaire for 88% of patients. CP and HL manually double checked locus of care, and contacts in secondary care on data entry forms to ensure data reliability.

### Analysis

We derived descriptive statistics relating to patient demographics, number and type of medications, number of co-morbidities, service contacts and reasons for contacts, and relational, cross boundary and informational continuity (see Box S1). Relational continuity was measured using the Modified Modified Continuity Index (MMCI) [29]. We have focussed on patients with poor continuity (see Box S1). We present data on these factors for patients with and without contacts with secondary care services (locus of care), and also broken down by mental illness diagnosis.

Direct statistical comparisons between patients with and without secondary care contacts were conducted using linear regression for continuous variables, poisson regression for count data (e.g. number of morbidities) and logistic/multinomial logistic regression for categorical variables, taking into account the clustering of patients within practices. We also conducted two further analyses to explore patient- and practice-level predictors of (i) locus of care and (ii) poor relational continuity. These took the form of multilevel (patients within practices) logistic regressions and were done in two steps: step one conducted a univariate analysis of each factor separately; step two combined all factors with a univariate p-value of p<0.2 in a multivariate regression.

All analyses were conducted using Stata version 11. The practice sample differed from practices nationally in being generally larger and less likely to be based in socioeconomically deprived areas. We therefore used probability weights in our analyses, based on each practice’s 2006/7 mental health register size (in national quartiles) and Index of Multiple Deprivation scores [30] (in national quintiles), to produce nationally representative estimates of patient percentages, means and measures of error (standard deviations and confidence intervals). All figures and statistical tests reported in the tables and text are weighted estimates, except where indicated. A significance level of 5% was used throughout.

## Supporting Information

Box S1 **Definition of concepts.**
(DOCX)

Box S2 **The data extraction form.**
(DOCX)
