# Supplementary material for: The Role of Primary Care in Service Provision for People with Severe Mental Illness in the United Kingdom
Source: PLoS One. 2012 May 15;7(5):e36468. doi: 10.1371/journal.pone.0036468 (PMC3352919; doi:10.1371/journal.pone.0036468)
Supplement: Box S1 — Definition of concepts. (DOCX) [file pone.0036468.s001.docx]

*Box S1 Definition of concepts*

| *Locus of care*  Management in secondary care was defined as fulfilling any of the following criteria: i) having a care coordinator; ii) being seen by a psychiatrist; iii) having a referral and contact with a secondary care service; or iv) having a contact with a secondary care professional (e.g. consultant/staff grade psychiatrist; CPN) during the year 1/4/08 – 31/3/2009. Any patient not fulfilling any of these criteria was defined as being seen only in primary care. The primary care group includes patients in contact with Day Care, Social Services, CBT, Occupational Therapy, MIND Day Centre, Community Day Centre or Private Services.  *Contacts with GP*  Face to face consultations during the year 1/4/08 – 31/3/2009 – not out of hours or telephone consultations. Up to four reasons for each contact could be recorded.  *Relational continuity of care*  Continuity of care was measured using the Modified Modified Continuity Index (MMCI):  =(1 - number of different GPs seen ⁄number of contacts with a GP) (1 - 1⁄number of contacts with a GP )  This measure relates a patient’s number of contacts with a health provider (e.g. GP practice) to the number of different professionals seen across those contacts (e.g. different GPs). In primary care, if all of a patient’s contacts were with the same GP then MMCI = 1; if they were all with different GPs then MMCI = 0.  *Poor relational continuity of care*  For this study we defined poor continuity of care as an MMCI of =<0.5. Mathematically this corresponds to a mean of less than two contacts for each different GP seen. We made an exception that three contacts across two GPs was not classed as poor continuity and did not compute an index for patients with less than 3 contacts in total.  *Informational continuity*  The timely availability of information. Measured by identifying patients who had a new referral to a mental health service over the year (1/4/08 – 31/3/2009): frequency of those patients with no information recorded in primary care about the outcome of the referral; those not seen by the mental health services; and those seen but where no documentation had been received by primary care.  *Cross-boundary continuity*  Effective communication between professionals: conceptualised as transitions and fragmentations. Measured by identifying those patients who were discharged from a mental health service during the year 1/4/08 – 31/3/2009: frequency of these patients who were either lost to follow up for no apparent reason or did not attend the appointment; those that did not have a reason recorded for their discharge.  *Dual diagnosis*  A current substance misuse or alcohol problem (as well as severe mental illness) recorded in the patient notes |
| --- |
