# Supplementary material for: The Role of Primary Care in Service Provision for People with Severe Mental Illness in the United Kingdom
Source: PLoS One. 2012 May 15;7(5):e36468. doi: 10.1371/journal.pone.0036468 (PMC3352919; doi:10.1371/journal.pone.0036468)
Supplement: Box S2 — The data extraction form. (DOCX) [file pone.0036468.s002.docx]

*Box S2 The data extraction form*

- Patient demographic details.
- Physical and mental health diagnoses.
- Current mental health medication at a single time point in 2009.
- Locus of care, all contacts with any staff member in primary and secondary care mental health services
- Reasons for contacts in primary care.
- All referrals for mental health and non mental health issues.
- All hospital admissions for any condition.
- All accident and emergency and walk in centre visits for any condition.
- Discharge details from mental health services.
- Detailed information on care co-ordinators and care programme approach meetings over a two year period (1/4/2007-31/3/2009).
- Practice details included list size, staffing structure, team development awards and training status and specific interest in mental health issues.
- Level of confidence in the data collected.
